# Optical pulse width modulation based TDM-PON monitoring with asymmetric loop in ONUs

**DOI:** 10.1038/s41598-018-22195-y

**Published:** 2018-03-14

**Authors:** Xuan Zhang, Xiaohan Sun

**Affiliations:** 1 College of Physics and Electronic Engineering, Sichuan Normal University, Chengdu, 610101 China; 2 National Research Center for Optical Sensing/Communications Integrated Networking, College of Electronic Science and Engineering, Southeast University, Nanjing, 210096 China

## Abstract

We propose a time division multiplexing passive optical network (TDM-PON) monitoring scheme based on the optical pulse width modulation (OPWM) by using a simple asymmetric loop (AL) located near the optical network unit (ONU). Various pulse widths can be easily obtained by configuring the appropriate length of the patchcord used to generate the path difference between two branches of a 1 × 2 splitter. Different wavelengths can be assigned to the same pulse width, which can further increase the network size. The optimized parameters of the AL are investigated and analysis models are established. The calculation results show that the proposed scheme has smaller correlation distance (CD) and lower multiple-customers interference probability (MCIP) than the periodic coding (PC) scheme. A simplified PON system with 8 ONUs is set up to experimentally demonstrate the feasibility of the proposed scheme. The network recognition processing used to identify the reflected monitoring signals in the proposed scheme is simple, especially compared with the reduced complexity maximum likelihood sequence estimation (RC-MLSE) used in the typical PC scheme.

## Introduction

Passive optical network (PON) will play key role in alleviating the last mile bottleneck for the next generation broadband optical access networks^1–3^. With the growth of optical fiber access networks and the expansion of broadband services, any service interruptions due to fiber cut may cause customers complaints and enormous loss in business^4–6^. Obviously, it is important for network operators to identify and fix the failure in the feeder fiber (FF) or drop fibers (DFs) timely in order to ensure the high quality of service (QoS)^7–9^. The fast fault detection can earn valuable time for the restoration, which reduces the probability of service disruption. Therefore, a simple but effective in-service monitoring technique is highly imperative for real-time fault identification along the fiber link^10–13^.

In the past decade, many schemes have been published in the scientific literature and others are available in the market for PON fault management. Each of which has its own advantages and disadvantages. Some of these schemes are already available in the market but with limited performance, and others are still under research or improvement. The performance of the scheme and overall cost of the monitoring system are two major research motivations. In addition, the monitoring scheme usually requires simple design, fabrication, and implementation procedures to reduce the cost^4,10^. Conventionally, optical time domain reflectometer (OTDR) is widely used to detect and locate a failure in point to point (P2P) network, but it is hard to play a role in point-to-multipoint (P2MP) or tree topology networks due to the superimposed backward signals from the different branches^14,15^. Several monitoring schemes have been proposed to overcome this problem. Reference reflector based monitoring scheme use wavelength selective reflectors at the end of each DF with tunable OTDR in the central office (CO). At the CO, the reflected wavelength of each DF is monitored and a fault can be detected from the presence or absence of this peak^16^. However, the network size (the number of network users in a PON system) of this scheme is often limited by the monitoring bandwidth because each DF has its own wavelength. Monitoring scheme based on Brillouin OTDR (B-OTDR) use a B-OTDR in the CO and DFs with unique Brillouin frequency shifts. Different DFs are distinguished from each other by the frequency of the peak in the spectrum of all Brillouin scattered light^17,18^. This scheme, however, requires manufacturing a different DF for each user, which calls for a dramatic change in current existing PON infrastructure making the cost extremely high. An optical in-service surveillance scheme generates both upstream data signal and surveillance signal by an upstream transmitter at each optical network unit (ONU), utilizing the self-injection locked reflective semiconductor optical amplifier (SL-RSOA)^19^. However, this scheme requires a protocol extension to avoid interference between data and monitoring wavelengths, which increase the complexity of the implementation procedures. The periodic coding (PC) scheme for PON monitoring provides a good solution with big network size and simple fabrication^20,21^. However, some challenges arise when deploying it in an actual network. For instance, correlation distance (CD) defined as the relative distance between two users after which their coding sequences cannot interfere with each other is poor, which leads to more difficult recognition process and severe multiple-customers interference probability (MCIP)^22^. The MCIP is defined as the probability that the intensity superposition of transmitted signals between multiple users and the desired user at the time slot. In addition, the reduced complexity maximum likelihood sequence estimation (RC-MLSE) as the network recognition algorithm requires much computational complexity due to the requirement of sufficient statistic^21^.

In this paper, we propose a time division multiplexing PON (TDM-PON) monitoring scheme by using the optical pulse width modulation (OPWM). Each ONU is assigned to a unique optical identification generated by the asymmetric loop (AL). Specifically, the identifications have the same wavelength but different pulse widths, or vice versa. That is, the proposed scheme can be clarified as a two-dimensional coding scheme. The calculation results show that the proposed scheme has better performance in CD and MCIP than the PC scheme. The network links status can be easily identified by the presence or absence of the pulse widths or wavelengths in most of the time. Note that the proposed scheme cannot provide the information of fault location or event type. However, it can monitor all DF links simultaneously in real-time way. The advantages of the proposed scheme mainly include 3 aspects: (1) The proposed scheme provides high network size monitoring because it can be encoded in two different dimensions (i.e., pulse width and wavelength). The ability to monitor high network size (64, 128 and beyond) makes the scheme applicable for next-generation PON (NG-PON). (2) The simple design of ALs not shared by the users can reduce the overall cost of the PON monitoring system. (3) The network recognition processing of the proposed scheme only needs to identify the pulse widths of different rectangular pulses, which is much simpler than the complicated network recognition algorithm used in most of PON monitoring schemes.

## Principle and Design

### Principle of Operation

**Figure 1:**
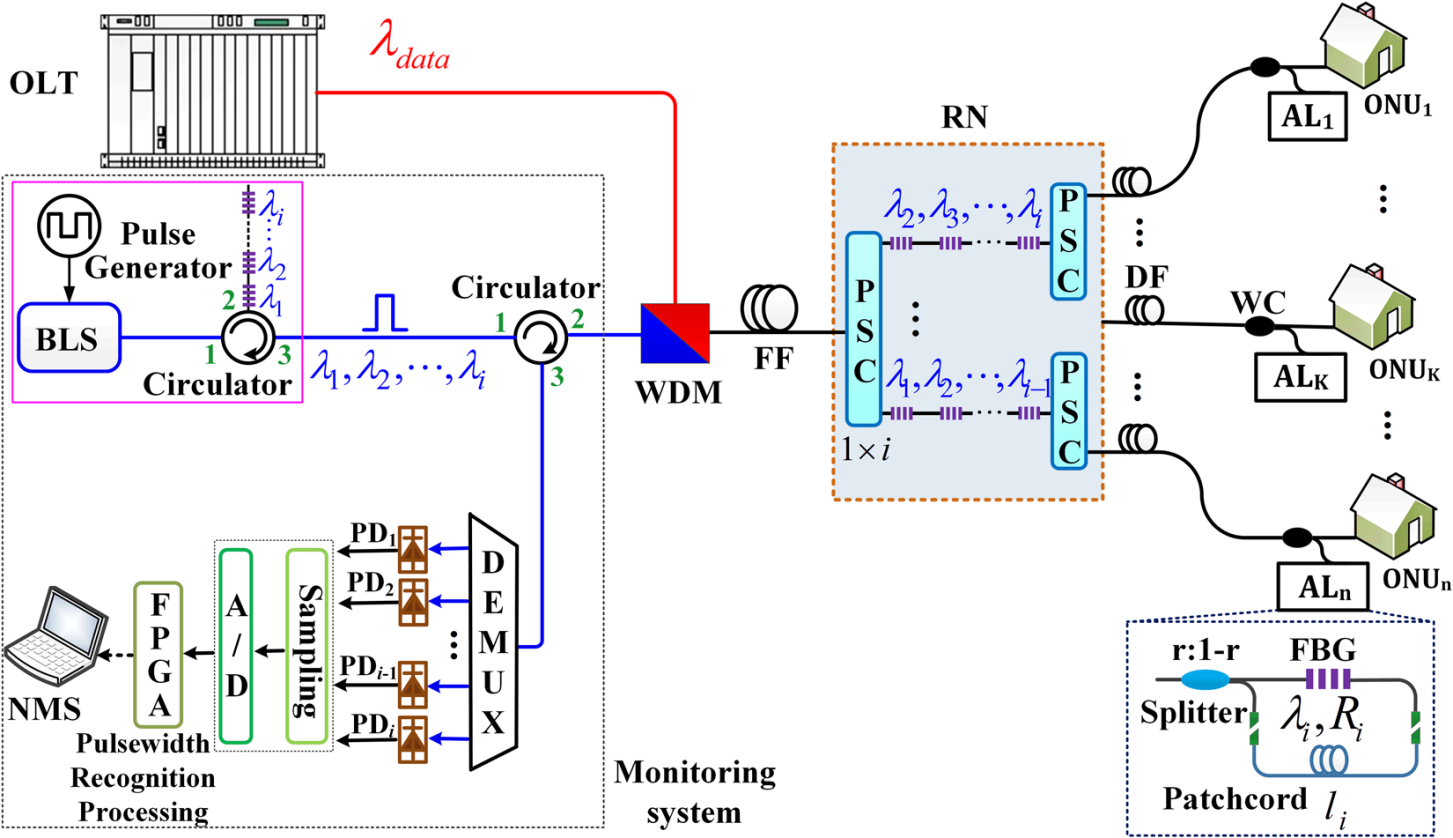
Principle of PON monitoring scheme based on the AL.

Figure 1 illustrates the principle of the AL based PON monitoring scheme. The rectangular detecting pulses with *i* ($i=1,2,\cdots$) wavelengths are synchronously injected into the network, which are extracted from the broadband light source (BLS) with direct modulation by using *i* tandem fiber Bragg gratings (FBGs). All the detecting pulses with an initial pulse width of ${T}_{s}$ in U-band (1625–1675nm, ITU-T G.983, reserved for standard PON monitoring) are coupled with normal data traffic in C-band via WDM coupler and then equally split into $n$ subpulses by the cascaded power splitter/combiners (PSCs) in the remote node (RN). The first and second stage connected through tandem FBGs in the cascaded PSCs respectively contain a PSC with *i* output ports and *i* PSCs with $n/i$ output ports, which can filter out all but the desired wavelength. Each subpulse is coupled by the identical wavelength coupler (WC) and broadened by the AL located near the ONU. Then, all the broadening signals are reflected back to the monitoring system, where the monitoring system is physically close to optical network terminal (OLT). Note that the cascaded PSCs can be replaced by a $1\times n$ PSC in single wavelength case. In the monitoring system, the return broadening signals are de-multiplexed to *i* wavelength channels by a wavelength division demultiplexer (DEMUX), followed by *i* photoelectric detectors (PDs). After the photoelectric conversion, the electronic signals are processed by the field programmable gate array (FPGA) through sampling and analog-digital conversion (A/D). The pulse width of each return signal can be identified after the pulsewidth recognition processing. Finally, the corresponding signals are sent into the network management system (NMS) to evaluate the network links status.

### Asymmetric Loop Design

As shown in the inset of Fig. 1, the AL is composed of a 1 × 2 splitter with coupling ratio $r$, a fiber Bragg grating (FBG) with the center reflected wavelength of ${\lambda }_{i}$ and reflectivity of ${R}_{i}$ and the patchcord with length of ${l}_{i}$. The patchcord is used to generate the path difference between two branches of the splitter. The overlapping area between the reflected pulses is intrinsically decided by the path difference. The pulse widths of the broadening sequences (composite pulses) output from the AL can be expressed as:

1 $$T\text{'}=\{\begin{array}{c}\,\,\,\,\,\,\,\,\,\,\,\,\,\,\,\,\,{T}_{s}\,\,\,\,\,\,\,\,\,\,\,\,\,\,\,\,\,\,\,\,\,\,\,\,\,\,\,\,\,\,\,\,\,\,\,\,\,\,\,\,\,\,\,\,\,\,\,\,{R}_{i}=0\\ \,{T}_{s}\,\,+\frac{2{n}_{g}{l}_{i}}{c}\,\,\,\,\,\,\,\,\,\,\,\,\,\,\,\,\,\,\,\,\,\,\,0\, < {R}_{i} < 1\,00 \% \\ \,{T}_{s}\,+\frac{{n}_{g}{l}_{i}}{c}\,\,\,\,\,\,\,\,\,\,\,\,\,\,\,\,\,\,\,\,\,\,\,\,\,\,\,\,\,\,\,\,\,\,\,\,{R}_{i}=100 \% \end{array}$$

where *c* is the speed of light in the vacuum, ${n}_{g}$ is the effective group index in the fiber core. ${T}_{s}$ and $T^{\prime}$ are the pulse width of the initial and broadening sequences, respectively. Note that the initial width ${T}_{s}$ of the detecting pulse determines the maximum length of the patchcord, which must satisfy the following restriction:

2 $${l}_{i}\le \frac{c{T}_{s}}{{n}_{g}}$$

Equation (2) ensures that only one pulse is contained in each broadening sequence, which greatly reduces the complexity of the final signal recognition.

**Figure 2:**
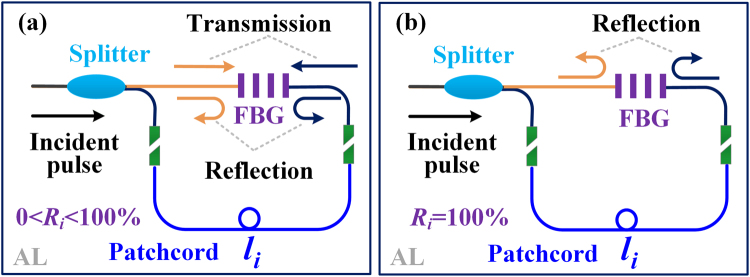
Operating principle of the ALs with different reflectivity. (**a**) $0 < {R}_{i} < 100 \%$. (**b**) ${R}_{i}=100 \%$.

As the pulse width is decided by the overlapping area of the reflected pulses, the coupling ratio *r* and the reflectivity ${R}_{i}$ determine the flatness of the broadening sequences. When ${R}_{i}$ is fixed to zero, the AL is transformed into Sagnac loop and lose the function of the OPWM. For $0 < {R}_{i} < 100 \%$, the incident pulse is injected into the AL and divided into three parts. The first part is reflected by the FBG firstly and the net path difference is zero. The second part contains two transmission subpulses, both of them experience path difference of ${l}_{i}$. The third part is reflected by the FBG lastly that experiences the path difference of double ${l}_{i}$. The operating principle of the corresponding AL with partial reflectivity is shown in Fig. 2(a). Obviously, the maximum broadening ratio reaches up to 3. Considering an incident pulse with the amplitude of *A*, we can obtain the amplitude of three parts ${A}_{1},{A}_{2},{A}_{3}$ from Equation (3):

3 $$\{\begin{array}{c}{A}_{1}={r}^{2}{R}_{i}A\\ {A}_{2}=2r(1-r)(1-{R}_{i})A\\ {A}_{3}={R}_{i}{(1-r)}^{2}A\end{array}$$

To keep the flatness of the broadening sequence, three subpulses are assumed to have the same amplitude. However, only at the maximum broadening ratio, the flatness of the composite pulse waveforms can be satisfied with $r=50 \%$ and ${R}_{i}=66.7 \%$. Note that the flatness of the composite pulse waveforms in the proposed scheme has no effect on the recognition processing, as the rising and falling edge of the first and last subpulse contributes to that. For the recognition convenience, symmetric pulse waveform may be expected. That is, the amplitude of the first and last subpulse must keep maximum simultaneously. To make ${A}_{1}$ and ${A}_{3}$ equal, *r* is fixed and only ${R}_{i}$ determines their amplitudes. In theory, larger amplitude is more conducive to the recognition. When ${R}_{i}$ is fixed to 100%, the incident pulse can be divided into two parts respectively reflected by the FBG. The path difference of between two parts is also double ${l}_{i}$. The operating principle of the corresponding AL with 100% reflectivity is shown in Fig. 2(b). Then, it seems to have the same expression of $0 < {R}_{i} < 100 \%$ in Equation (1). Note, however, that the broadening ratio must be less than 2 because only one pulse is contained in each broadening sequence. If we take the same expression for two different ${R}_{i}$, the patchcord length ${l}_{i}$ must be distinguished, otherwise the maximum broadening ratio for ${R}_{i}=100 \%$ may be 3 when ${l}_{i}$ is equal to $c{T}_{s}/{n}_{g}$. Taking into account the universality of ${l}_{i}$, we take this expression of ${R}_{i}=100 \%$ in Equation (1) as all values of allowed broadening pulse widths fall within it. From Equation (3), only the coupling ratio *r* decides the flatness and $r=50 \%$ ensures the best recognition value. Considering the bigger broadening ratio, we mainly focus on the case of $0 < {R}_{i} < 100 \%$ in the later discussion.

## Analysis and Results

The system performance of the proposed scheme is evaluated by the CD and MCIP. Based on the previous principle and design of the proposed scheme, we build corresponding analysis models and give the calculation results. In addition, we also give some analysis of the network recognition processing used in the proposed scheme.

### Correlation Distance

**Figure 3:**
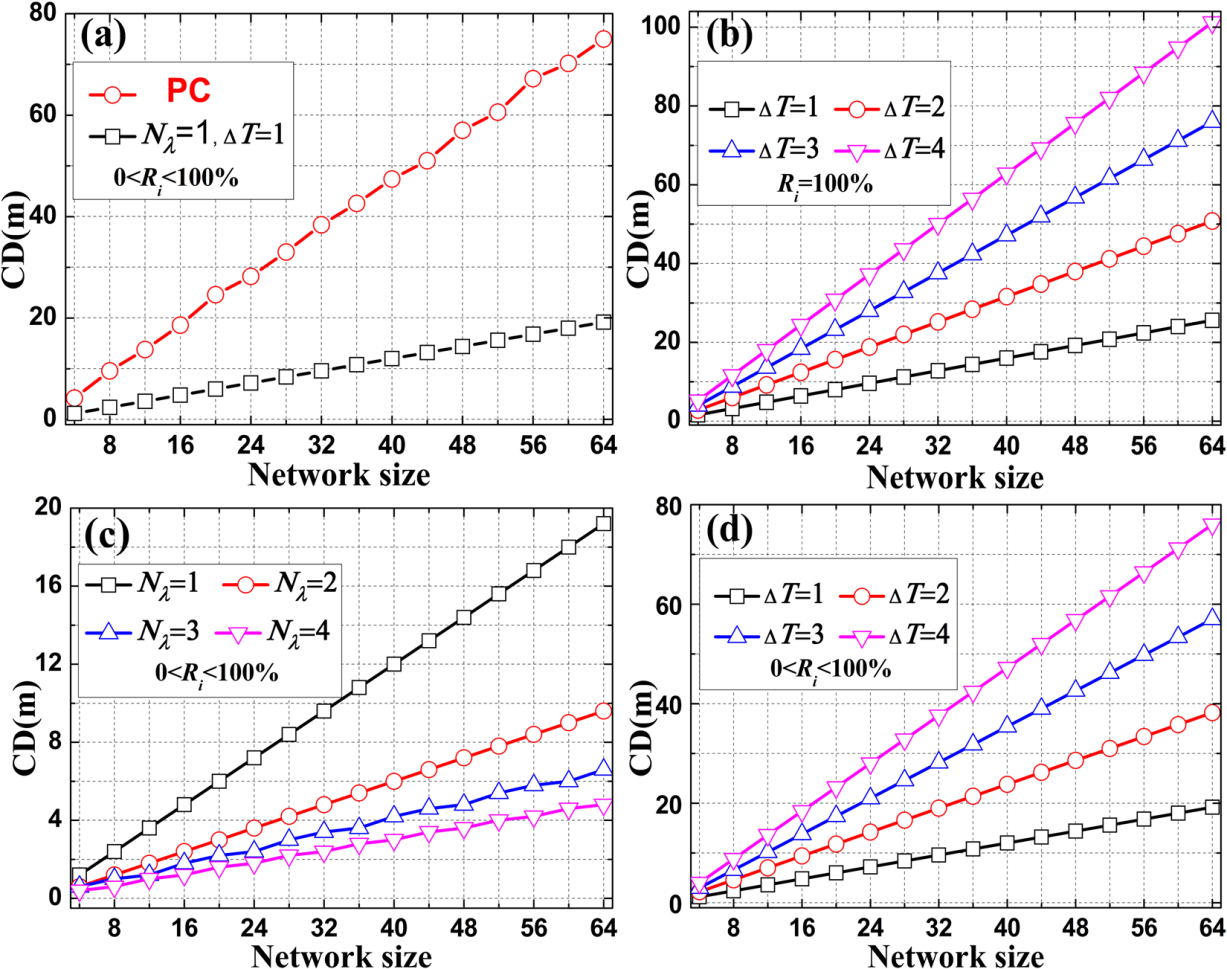
Correlation distance varies with the network size. (**a**) The PC scheme and the proposed scheme with ${N}_{\lambda }=1$ and ${\rm{\Delta }}T=1$. (**b**) Single wavelength corresponding to various ${\rm{\Delta }}T$ with ${R}_{i}=100 \%$. (**c**) ${\rm{\Delta }}T=1$ corresponding to various ${N}_{\lambda }$ with $0 < {R}_{i} < 100 \%$. (**d**) Singe wavelength corresponding to various ${\rm{\Delta }}T$ with $0 < {R}_{i} < 100 \%$.

As we all know, any physical separation between two users less than the CD may interfere with each other and then contribute to MCI. In the proposed scheme, the monitoring signals with the same wavelength are assigned to different pulse widths. For single wavelength, higher network size requires more monitoring signals with different pulse widths, and then leads to bigger pulse width. Consequently, greater CDs may increase the mutual interference probability between users and the false alarm rate (FAR) of the network recognition processing. Recall that the proposed scheme is a two-dimensional coding scheme. Hence, MCI can be eased substantially in multi-wavelength case. In such circumstances, multiple wavelengths are allowed to be shared with the same pulse width, which effectively reduces the CDs. Based on above analysis, the CD in the proposed scheme can be written as:

4 $${L}_{CD}=\frac{c\cdot [{T}_{0}+(\lceil n/{N}_{\lambda }\rceil -1)\cdot {\rm{\Delta }}T]}{{n}_{g}}$$

where ${T}_{0}$ is the minimum pulse width in the broadening sequences, *n* is the network size, ${N}_{\lambda }$ is the number of wavelengths, $\lceil \rceil$ is the ceiling function, whose value is the smallest integer not less than $n/{N}_{\lambda }$, ${\rm{\Delta }}T$ is the step size of any two adjacent pulse widths. Consider all the pulse widths assigned to users in nanosecond. Here, we choose ${T}_{0}={T}_{s}+1$ to calculate the CDs under various conditions, as shown in Fig. 3. In Fig. 3(a), the CDs for the PC scheme and the proposed scheme corresponding to a PON with 64 users are 75 m and 19.2 m, respectively. In the calculation, ${T}_{s}$ is set to 32 *n*s when the FBGs of the ALs are partial reflectivity. The minimum and maximum patchcord length are 6.6 m and 19.2 m, respectively. Note that smaller ${\rm{\Delta }}T$ can further reduce the patchcord length. If we regard the broadening sequences with different pulse widths as optical orthogonal codes used in the PC scheme, the pulse width between the first and last falling edge of the broadening sequence can be regarded as the code length. According to the definition of the CD in the PC scheme, it is closely related with the code length of a code. Obviously, all the codes of the proposed scheme are much more compact than that of the PC scheme in the time domain, which leads to a smaller CD. From the rest of subplots, we conclude that big step size lead to greater CDs and more wavelengths lead to smaller one. The CD corresponding to $0 < {R}_{i} < 100 \%$ is superior to ${R}_{i}=100 \%$. In Fig. 3(b), ${T}_{s}$ corresponding to 4 different step sizes with ${N}_{\lambda }=1$ and ${R}_{i}=100 \%$ are 64 *n*s, 127 *n*s, 190 *n*s and 253 *n*s, respectively. When ${\rm{\Delta }}T$ is set to 1 *n*s, ${T}_{s}$ corresponding to 4 different wavelengths with $0 < {R}_{i} < 100 \%$ are respectively 64 *n*s, 127 *n*s, 190 *n*s and 253 *n*s, the calculation results are shown in Fig. 3(c). In Fig. 3(d), ${T}_{s}$ corresponding to 4 different step sizes with ${N}_{\lambda }=1$ and $0 < {R}_{i} < 100 \%$ are 32 *n*s, 64 *n*s, 95 *n*s and 127 *n*s, respectively.

### Multiple-Customers Interference Probability

**Figure 4:**
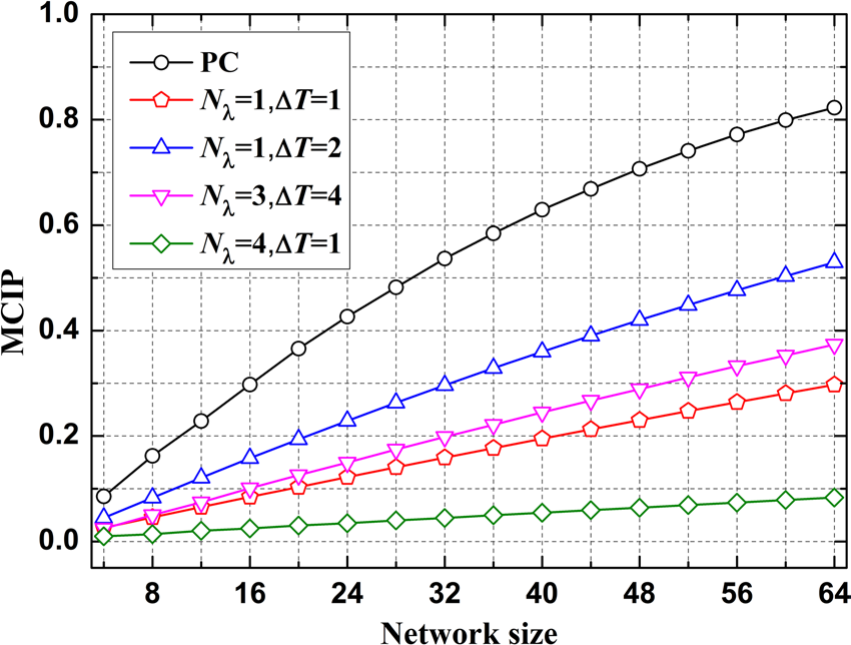
MCIP varies with the network size for the PC scheme and the proposed scheme with various wavelengths and step sizes.

As mentioned earlier, the MCI is closely related to the CD, which occurs when any two pulse waveforms contact with each other. Note, however, that the overlapping waveform can also be processed in the electronic domain but not covered here. In the proposed scheme, the MCI is mainly caused by the same wavelength with different pulse widths due to the co-channel interference. The same pulse width, even corresponding to the same DF link, never interferes with each other because the pulse widths with various wavelengths are recognized electrically from different channels. Furthermore, the same wavelength never appears twice, thus not contributed to the MCI in the frequency domain. Hence, the MCIP can be calculated as:

5 $$P(MCIP)=\frac{1}{n-1}\sum _{i=1}^{n-1}\sum _{j=i+1}^{n}{P}_{I}(2|{l}_{i}-{l}_{j}|\le \,\max ({L}_{CD}(i),{L}_{CD}(j)))$$

where *i* and $j$ denote any two users, ${l}_{i}$ and ${l}_{j}$ are the length of the respective DF links. *n* is the network size. The length difference between ${l}_{i}$ and ${l}_{j}$ is doubled due to the round-trip path of the monitoring signals. In the proposed scheme, each user is assigned to a unique pulse width at the dedicated wavelength. Here, we take Monte Carlo simulations with random assigned pulse widths and any location in a PON at 10^5^ times iteration to calculate the MCIP. The users are considered to be randomly distributed over a 5000 m^2^ coverage area. As is shown in Fig. 4, the MCIP of the PC scheme is 0.82 in a PON with 64 users while the MCIP of the proposed scheme corresponding to the single wavelength with 1 *ns* step size is roughly 0.3. Obviously, ${T}_{s}$ and the minimum patchcord length are 32 *n*s and 6.6 m, respectively. For 4 wavelengths with 1 *n*s step size, the MCIP is just 0.08. Here, ${T}_{s}$ can be set to 8 *n*s, supporting 16 users in single-wavelength case. Thus, the minimum patchcord length is 1.6 m. Single wavelength with 2 *n*s step size (${T}_{s}$ = 64 *n*s) and 3 wavelengths with 4 *n*s step size (${T}_{s}$ = 43 *n*s) are 0.53 and 0.37, respectively. As mentioned before, the broadening sequences are regarded as the optical orthogonal codes in the PC scheme, which can generate small code length and then leads to a smaller CD. As a consequence, the system performance (i.e., CD and MCIP) can be improved due to the codes with smaller CDs for the users.

### Network Recognition Processing

For current popular PON monitoring schemes, MLSE is the most plausible option to be the network recognition algorithm. However, due to the uncertainty of the geographical distribution, the network recognition algorithm must provide all possible network links status beforehand. With the PC scheme, for example, a PON with only 8 users, the maximum separation length of 17.8 m (1 km^2^ coverage area) and a receiver sampling rate of 1Gsps requires 178^8^ comparisons for the MLSE. The computational amount increase dramatically with the increase of the network size and coverage area. Obviously, the MLSE and modified RC-MLSE are inefficient and time-consuming network recognition algorithm. In the proposed scheme, the transmitted detecting signals are rectangular pulses and the initial pulse widths are decided by the network size. Consequently, all identifications with unique pulse widths for users are real numerical values. The recognition processing is a binary decision that compares the hypothesized values with the measured values and determines whether the two match. It is irrelevant to the unpredictable length of each DF link. The DF link length determines the comparing order of different pulse widths but not affects the final recognition of the network links status. Also for a PON with 8 users, only 36 comparisons are required in the proposed scheme. The first comparison is 8 times, the second can be reduced to 7 times, and the last need only 1 time. That is, once we compare a value with the measurements and find it, then no need to compare other values with the one already found. The missing value indicates that a break occurs in the corresponding DF link. Note that it is easy for the FPGA to recognize a rectangular pulse width by capturing the difference of the time of arrival (TOA) between the rising and falling edge. The pulse width measurement technology based on the FPGA has been widely developed and the implementation is easy. Compared with the RC-MLSE used in the PC scheme, the network recognition processing in the proposed scheme is simple. Due to the smaller CD or MCIP, all network links status can be easily identified in most of the time. When the signals with MCI appear in the return signals, the amplitude information of return signals is also used to distinguish between the signals with interference. The difference between pulse amplitudes can be obtained by using high-speed sampling in the overlapping area.

### Experimental demonstration

A simplified PON with 8 ONUs are selected for the experimental demonstration. Taking account into the equipment availability, we use the super-luminescent light emitting diode (SLED) in C-band with direct modulation to generate the periodic detecting pulses with pulse width of ${T}_{s}=100\,ns$ and repetition rate of ${T}_{P}=1\,kHz$. The detecting pulses with two wavelength chips of ${\lambda }_{1}=1549.3\,nm$ and ${\lambda }_{2}=1550.9\,nm$ are spectrally sliced by two tandem FBGs with the reflectivity of 95%. Erbium doped fiber amplifier (EDFA) is employed to compensate the power loss. The detecting pulses launch into a 20 km single mode fiber via an optical circulator and then reach the RN. In the RN, the detecting pulses are power splitted and filtered by two FBGs with the center reflected wavelengths of ${\lambda }_{1}$ and ${\lambda }_{2}$, respectively located at two branches of 1 × 2 PSC and followed by two 1 × 4 PSCs. The subpulses are broadened and reflected back by the respective ALs at each ONU. Total broadening signal is de-multiplexed by a DEMUX and then sent to the PDs. Finally, the measured traces are captured by a real-time oscillograph.

**Figure 5:**
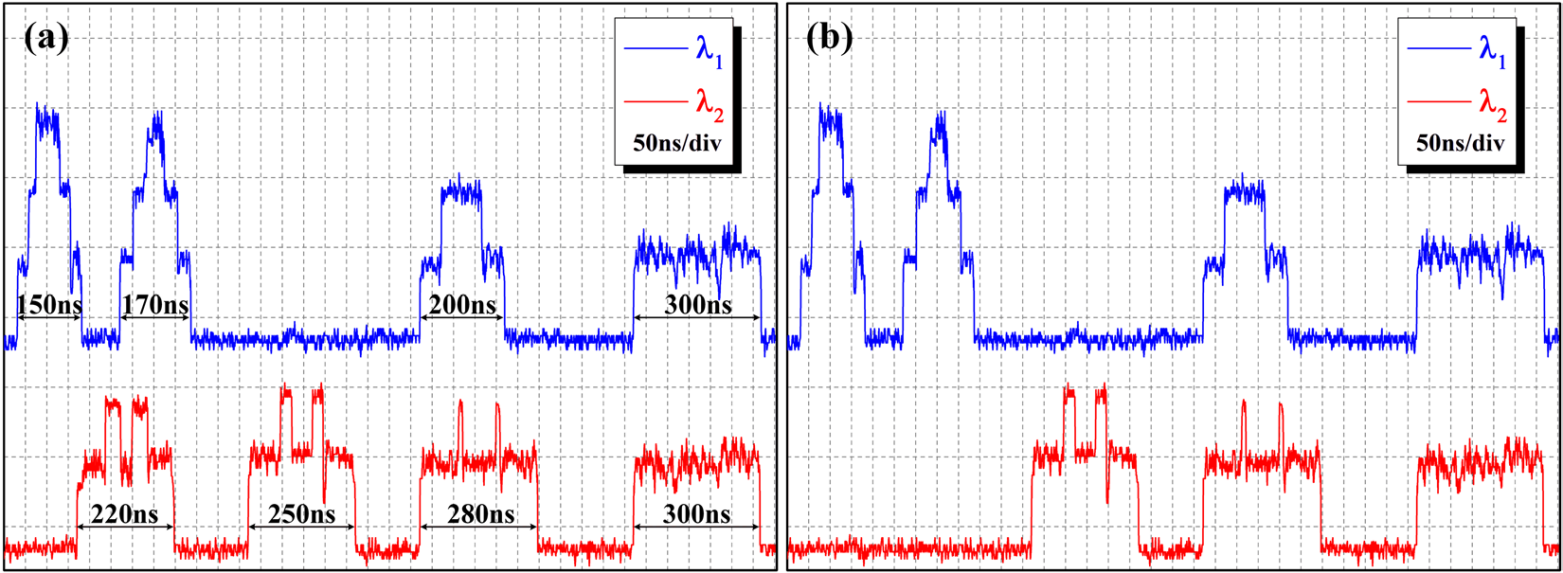
Experiment results. (**a**) The demultiplexed signals with 8 DFs in the healthy links condition. (**b**) A break of DF_5_.

The center reflected wavelengths of FBGs and patchcord length of 8 ALs are (${\lambda }_{1},{l}_{1}=5\,m$), (${\lambda }_{1},{l}_{2}=7\,m$), (${\lambda }_{1},{l}_{3}=10\,m$), (${\lambda }_{1},{l}_{4}=20\,m$) and (${\lambda }_{2},{l}_{5}=12\,m$), (${\lambda }_{2},{l}_{6}=15\,m$), (${\lambda }_{2},{l}_{7}=18\,m$), (${\lambda }_{2},{l}_{8}=20\,m$), corresponding to the DFs from 1 to 8 in turn. Note that the subscripts between DF and ONU are in one-to-one correspondence. The 3 dB bandwidth and reflectivity of FBGs are roughly 0.25 nm and 67%, respectively. Waveform traces for two wavelengths with different pulse widths are shown in Fig. 5. Recall that the broadening sequence keeps flatness at the maximum patchcord length, corresponding to 20 m in this experimental demonstration. Figure 5(a) shows the waveforms of two wavelengths with 7 different pulse widths in the healthy case. The same pulse width of 300 *n*s is configured for different wavelengths ${\lambda }_{1}$ and ${\lambda }_{2}$ indicate that wavelengths can be used as another available dimension, which proves the concept of the two-dimensional coding scheme. The measured values of the broadening sequences are labeled on the corresponding monitoring signals, being in full agreement with Equation (1). In Fig. 5(b), a missing pulse waveform of ${\lambda }_{2}$ with pulse width 220 ns indicates a break in the DF_5_ link.

## Conclusion

We have proposed and experimentally demonstrated OPWM in the optical domain by using the ALs for TDM-PON monitoring. The design of ALs with different reflectivity is given and the relatively optimized parameters are investigated. The calculation results show that the proposed scheme provides better performance in terms of CD and MCIP than the PC scheme. Monitoring signals can be generated by using pulse widths and wavelengths simultaneously, which is well suited for high network size. The simple network recognition processing provides great convenience for the evaluation of the network links status. The two- dimensional coding offers a promising solution for monitoring of future high capacity PONs.
